# Evaluating eHealth: How to Make Evaluation More Methodologically Robust

**DOI:** 10.1371/journal.pmed.1000186

**Published:** 2009-11-24

**Authors:** Richard James Lilford, Jo Foster, Mike Pringle

**Affiliations:** 1 Division of Primary Care, School of Health and Population Sciences, University of Birmingham, Birmingham, United Kingdom; 2 School of Community Health Sciences, University of Nottingham, Nottingham, United Kingdom; Edinburgh University, United Kingdom

## Abstract

In the third in a series of articles on evaluating eHealth, Richard Lilford and colleagues consider the evaluation of health IT systems as they are rolled out following preimplementation testing.

*This is the third in a series of three articles on evaluation of eHealth.*

**Summary Points**

- Evaluation of information technology (IT) systems often requires a mixed methods approach.
- External evaluations have many advantages, especially in terms of standardisation, independence, and the possibility of using controlled before and after designs.
- Difficulties arise when commissioners ask external evaluations to also provide formative assessments designed to assist in the implementation itself. Under these circumstances the summative results, which encapsulate the overall benefits and harms of a system, may be rendered less generalisable.
- We think researchers and commissioners should resist the current fashion of asking external academic teams to combine formative with summative assessments.

eHealth—the organisation and delivery of health services and information using information technology (IT) systems—is playing an increasingly important role in shaping health care systems. However, as Catwell and Sheikh described in the first article in this series [1], IT systems can introduce harms as well as benefits. Catwell and Sheikh argued for a general scheme of evaluation starting with careful specification of need and pre-implementation testing in their article. This philosophy of pre-implementation testing resonates strongly with the UK Medical Research Council (MRC) framework for evaluation of complex interventions [2], the tenets of safety science (which endorses the use of analytic procedures to predict the failure rate of a system still in the design phase), and established principles in the IT field where “alpha testing” is routine. But how should IT systems be evaluated as they are rolled out following pre-implementation testing? This is the aspect of eHealth we will consider in this essay.

## eHealth in the UK

Our approach to the evaluation of eHealth has been strongly influenced by the roles we have played in commissioning evaluation research on behalf of the National Programme for Information Technology (NPFIT), an initiative by the Department of Health in England to move the English National Health Service (NHS) towards a single, centrally mandated electronic care record for patients and to connect general practitioners to hospitals. The NHS is investing several billion pounds sterling each year in IT. Investment in a programme of evaluation alongside the NPFIT programmes has provided an excellent opportunity to identify newly installed IT systems and to commission prospective studies to assess these programs as they are rolled out. Such evaluations fulfil a real need, since a recent systematic review showed that “most of the high quality literature regarding multifunctional health information technology systems comes from 4 benchmark research institutions” and that “little evidence is available on the effect of multifunctional commercially developed systems” [3] such as those that are increasingly implemented in the NHS.

Because these evaluations would inevitably be commissioned under intense political and media attention, NPFIT took the principled decision to contract the University of Birmingham to commission the research independently under Department of Health procurement rules. NPFIT could thus influence what was commissioned (i.e., research topics) but not the results obtained.

All research commissioners have to take some responsibility for determining the form that research takes. This is particularly so when, as in the case of the evaluation of NPFIT, it is the research commissioner, rather than the researcher, who puts the ball in play. It is the research commissioner who specifies what is to be researched, over what time scale, and with what level of resource. During the course of commissioning evaluation studies for NPFIT, we identified four tricky issues that we think both commissioners of eHealth research and eHealth researchers need to consider, namely: (1) which research methods are suitable for the evaluation of highly complex interventions with diffuse effects, such as IT systems; (2) whether it is necessary to make observations at both the patient and the system level; (3) whether to conduct research that strengthens or improves the intervention being evaluated (formative research) and/or research that examines the benefits or outcomes of that intervention (summative research); and (4) whether to evaluate research both externally and internally.

## The Case for Multiple Methods Research

There is a consensus about the evaluation of clinical treatments, such as drugs in which randomised control trials are state of the art. No such consensus exists yet for the evaluation of highly complex service interventions such as computer systems. However, we believe that the best way to evaluate eHealth is through “methodological pluralism” [4]–[8]. That is, research commissioners and research teams need to recognise the importance of undertaking combined quantitative and qualitative work when evaluating IT systems. Quantitative research can provide important numerical information about how IT systems are performing and is important in theory building, which is necessary to understand how interventions work (not just whether they worked in a particular set of instances) and hence to inform judgements about the generalisability of results from one context to another. Qualitative research can provide information on topics such as ease of use, which will ultimately affect whether the IT system is successful. So, for example, quantitative data may show that computer decision support has little impact on clinical error, while qualitative work explains why—for instance, clinicians may experience alert fatigue. More controversially qualitative research can also contribute to parameter estimation, particularly under a Bayesian framework [9]. More detailed accounts of methodological pluralism can be found elsewhere [5]–[8],[10]–[13].

## Observations at Patient and System Level

Although IT systems can sometimes be studied at the level of individual patients (e.g., computerised decision support) [14]–[16], they often need to be studied at the organisational level [17]. In some cases, this is because IT systems simply cannot be restricted to certain individuals in a group (for example, a computerised theatre booking system). In other cases, “contamination” (where an intervention “leaks” from a person in an intervention group to one in a control group) may dilute any positive effects at the individual level [18]. The primary unit of analysis in evaluation of IT systems is therefore likely to be at the organisational/workgroup level (e.g., wards, hospitals, practices). In this respect, IT assessments frequently have much in common with other forms of Service Delivery and Organisation research [19].

**Figure 1:**
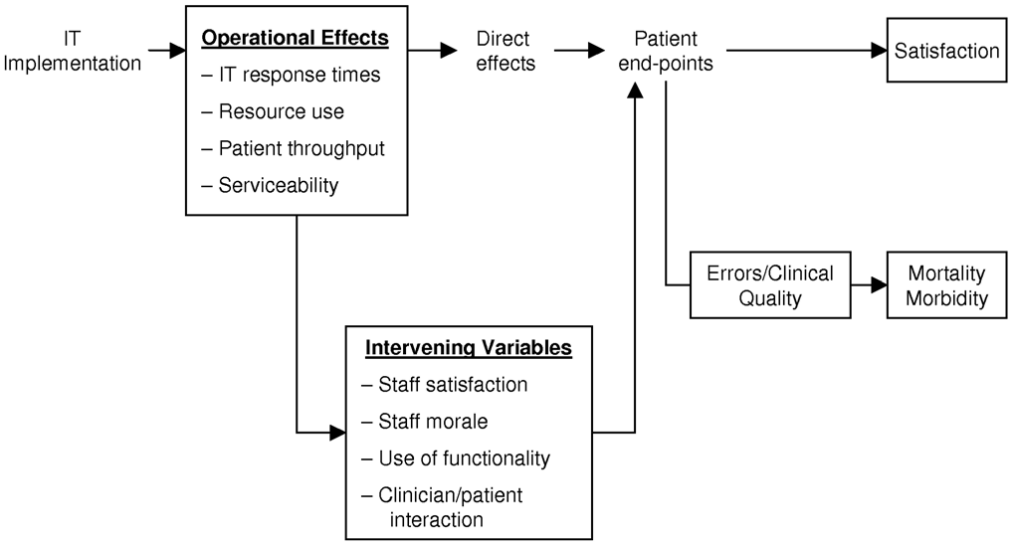
Causal chain showing levels where IT may impact. The potential impact of IT at different levels in a health care organisation. These boxes show endpoints that can be measured at different stages of the causal pathway. These endpoints include system effects (operational effects), effects on mediating variables, and endpoints at the patient level such as clinical errors and their sequelae.

Like other system-based interventions, IT may impact at many levels in the organisation and may have many effects (good or bad) at these different levels [20]. We have conceptualised these levels in the form of a causal chain (Figure 1). IT interventions, like other patient safety and service delivery interventions, frequently need to be studied at all of these levels [21] for several reasons. First, data of different types can be collated from all points on the causal chain to provide information not just on what happened (e.g., to what extent did prescribing improve) but on why it happened (e.g., did clinicians over-ride computer generated suggestions, and if so, why?). Computer systems may have negative influences on clinical consultations [22],[23] and time efficiency [24], and may encounter cultural barriers [25], or be affected by broader political forces. Feedback from decision support systems is frequently ignored. All these problems need to be tackled, and collection of information at many levels is necessary to generate theories about possible explanations and remedies. For example, in-depth studies have shown that the psychological effects of clinical computer systems are more positive when both patient and clinician can view the computer screen [26].

Second, the robustness of research findings is increased if a service delivery implementation, such as an IT installation, has impacted positively at many levels in the causal chain; consistent change in endpoints acts as a form of “triangulation.” That said, it should be emphasised that the final purpose of an IT system is to impact positively on the patient. The various endpoints at the service level may be necessary, but not necessarily sufficient, conditions for a positive impact at the patient level. Third, multiple measurements across the chain, including time-efficiency, throughput, and patient satisfaction are all necessary to model cost-effectiveness/cost-benefit. Finally, base-line observations across the chain provide evidence on context. For example, failure to find improvement in clinical processes is likely if practice is already very good. Likewise, prevailing attitudes in an organisation can undermine a computer system as seen at the Cedars Sinai Hospital in California [27].

Impact at the patient level (Figure 1) can be divided into the patient experience (including satisfaction), mortality, and morbidity. An effect on mortality and morbidity may not be observed, even if the implementations are effective (i.e., there is a high chance of a false negative result). This high risk of a false negative results arises because the signal-to-noise ratio is high when mortality and morbidity are used as measures of service quality [28]. For this reason, it is important to collect error rates/clinical process data wherever possible. Such errors are typically much more prevalent than the outcomes they portend. The topic of measurement of clinical processes/errors has been explored in detail elsewhere [5]–[8],[10],[29]. One particular problem with IT, however, is that the intervention and measuring systems are not necessarily independent. For example, replacing clinical notes with an IT system substitutes both the platform for delivery of care and the platform for measuring the quality of that care.

## Formative and Summative Assessment

By formative assessment, we mean studies that provide timely feedback to those who are responsible for implementing an IT system [30],[31]. By summative assessment, we mean the provision of generalisable knowledge that will inform decision makers elsewhere and into the future, well beyond the life cycle of a particular application. Clearly these are not watertight distinctions. Well-conducted formative studies are seldom totally devoid of generalisable lessons, even those in which feedback is specific and immediate [32]. Summative research, on the other hand, tends to produce results over a longer time scale and its purpose is often directed at future IT implementations rather than at the projects that were the subject of study.

Can researchers and managers have their cake and eat it by combining summative and formative research into single research projects? Our answer is only a cautious “yes.” Some of the results of assessments typically become available earlier than others. For example, effects on intermediate variables such as staff acceptability tend to be available earlier than the results of time series analyses of error rates. However, if the results of formative research are fed back into implementations, this may influence summative results. In circumstances where formative research will not be part and parcel of future implementations, summative research with formative research nested within it may yield greater estimates of benefit than future implementations shorn of a formative component.

Because external evaluation will not necessarily be included in future implementations, we would prefer a world in which external assessors carry out summative rather than formative assessments. However, the funders of interventions may expect or demand feedback of interim data to inform implementations. Clearly, there is tension here—in our opinion a tension that is too often wished away under the banner of subjectivist/interpretivist research.

A pragmatic policy for research commissioners under these circumstances is to commission both formative and summative assessments from the research team, but to try to identify any effects of the former on the latter. To do this it is necessary to track developments over time at intervention and control sites, noting whenever formative results are fed back to the implementation teams. The hypothesis to be tested is that improvements are incremental and then become stable over time, suggesting that the lessons of earlier applications have been incorporated in successive applications. The step-wedge design [33]—a design in which later adopters of an IT system act as controls for early adopters—seems to have particular promise in the evaluation of healthcare IT systems in that it has logistic and certain scientific advantages over standard parallel designs. The largest project commissioned under the NPFIT evaluation programmes (an evaluation of comprehensive hospital-based IT systems as they are rolled out) follows a step-wedge design, although as a “natural experiment” rather than randomised trial.

## External and Internal Assessments

An element of formative assessment is a tenet of good implementation policy and thus some formative internal evaluation should be intrinsic to IT implementations. Such an evaluation lies outside an externally sponsored research programme. Any formative internal evaluation carried out by the implementation team should be described, along with other features of the IT system and its context, as with any complex intervention. However, external assessments add value to the evaluations carried out in-house by the implementation teams.

External assessment can provide expertise in the measurement of endpoints (for example, error rates/quality) where special expertise is needed. In quality measurement many methodological traps lie in wait for nonexperts [6],[28],[29]. For instance, internal assessors may be (subconsciously) biased in measuring outcomes and/or they may have different levels of performance over time—learning or fatigue effects. External assessors can be masked with respect to time, place, and the hypothesis, and error rates from different epochs can be reviewed in parallel so that any learning effect can be allowed for. External assessment can also bring in contemporaneous control observations, thereby helping to distinguish between temporal trends and causal associations [7]. Wherever possible before and after controlled studies should be used to reduce bias [7]. Finally, external assessment is independent from the implementation teams and hence credible to a wider audience.

**Figure 2:**
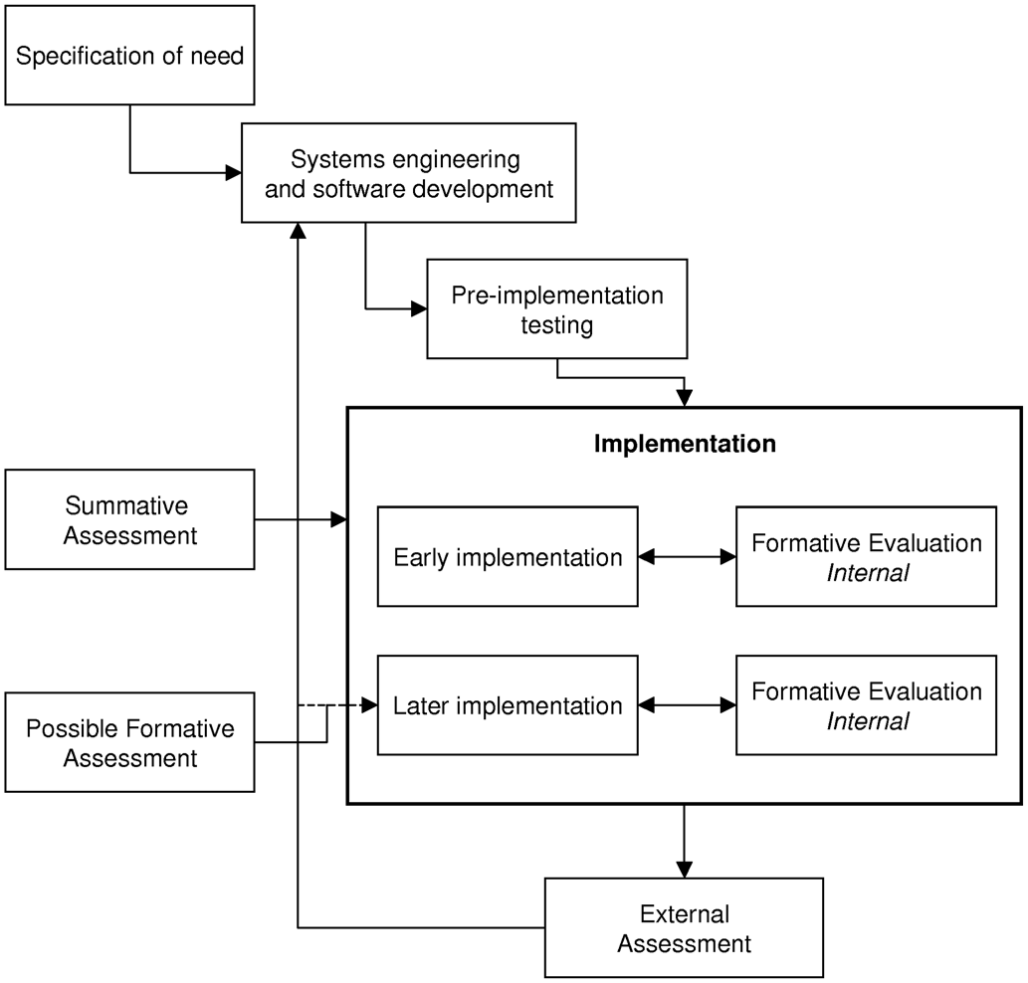
Diagrammatic representation of development and deployment of IT systems. Diagrammatic representation of the development and deployment of IT systems to demonstrate the ideas of internal versus external assessment on the one hand and formative versus summative evaluation on the other.

Figure 2 shows an idealised scheme depicting the development and deployment of IT systems in healthcare. Here, we illustrate the concept of formative versus summative assessment on the one hand and internal versus external assessments on the other. These types of assessments sometimes have to be combined, but we would prefer to separate them into two different paradigms: (1) formative assessments that are carried out by internal teams (or through collaborations between internal teams and “consultants”); and (2) summative assessments that are independent, operate over larger time frames, are conducted by disinterested “academic” teams, and incorporate both before and after measurements and external controls. Finally, Figure 2 also draws attention to our belief that more attention should be given in guidelines for reporting evaluation studies in health informatics to the potential effect of feedback during the course of a study [34].

## Conclusions

In this essay we have articulated a set of scientific principles for evaluating highly complex service interventions such as IT systems. In doing so, we have stuck closely to accepted scientific principles and have attempted to show that, with appropriate care, these principles can be incorporated into the evaluation of major service delivery interventions in the real world.

The programme we have put in train to evaluate NPFIT broadly speaking embodies these principles and has managed to track interventions prospectively, thus fulfilling its main aim. The topics of investigation included in our evaluation programme are (quite properly in our view) heavily influenced by NPFIT. However, the findings of the programme are independent of NPFIT in the sense that NPFIT cannot suppress or alter them. Most, but not all, of the commissioned research was of a purely summative nature. More generally we discern a growing pressure across all service delivery evaluation projects to ask researchers to be “all things to all people” and provide both formative research for service managers and scientific data with international significance, all in the same study. We argue against this tendency. However, since managers understandably want short-term results to help with implementations rather than longer-term results to provide new knowledge for the world as a whole, they may decide, to take their cheque book elsewhere. This is a price the authors of this essay would willingly pay to ensure that the evaluation of eHealth and other complex service interventions is as robust as possible.
